# A Study on the Factors Associated with College Students’ Intention to Use AI-Based Mental Health Services: The Roles of Trust, Empathy, and Privacy Concern

**DOI:** 10.3390/bs16101874

**Published:** 2026-10-11

**Authors:** Meng Fan, Xiaowei He, Mengyuan Zhou, Tiansheng Xia

**Affiliations:** 1 Mental Health Education Center, Guangdong University of Technology, Guangzhou 510006, China; 2 School of Art & Design, Guangdong University of Technology, Guangzhou 510090, China

**Keywords:** artificial intelligence, mental health services, higher education, technology acceptance model, trust, privacy concern, digital mental health

## Abstract

Artificial intelligence technology is gradually being integrated into mental health service contexts and is increasingly discussed as a potential supplementary resource to university mental health support systems. However, AI-based mental health services involve sensitive factors such as emotional expression, personal privacy, and relational interaction. Therefore, college students’ willingness to accept AI-based mental health services and the potential factors associated with such acceptance remain unclear. Based on an extended Technology Acceptance Model, this study introduces six factors—Accessibility (ACC), Privacy Concern (PC), Perceived Anthropomorphism (PA), Perceived Empathy (EMP), Trust (TRU), and Perceived Usefulness (PU)—to systematically examine their relationships with university students’ Behavioral Intention (BI) to use AI-based mental health services. A total of 375 volunteers participated in the current study and completed relevant questionnaires. Structural equation modeling was employed for data analysis. The results indicate that accessibility, perceived usefulness, and trust are all significantly and positively associated with behavioral intention. Perceived empathy is significantly and positively associated with trust, whereas privacy concern is significantly and negatively associated with both trust and behavioral intention. The analysis of indirect effects shows that there is a significant sequential indirect association between perceived anthropomorphism and behavioral intention through perceived empathy, trust, and perceived usefulness. In addition, a significant negative indirect association between privacy concern and behavioral intention through trust was observed. The findings indicate that, in the highly sensitive context of mental health services, college students’ intention to use AI-based mental health services is associated with a combination of accessibility, perceived usefulness, emotional interaction experience, privacy-related evaluations, and trust.

## 1. Introduction

In recent years, artificial intelligence technology has increasingly entered the field of mental health services and has gradually become an important force driving innovation in mental health care (Olawade et al., 2024). In this study, AI-based mental health services is used as an umbrella term to refer to AI-enabled digital tools used for mental health-related purposes, including emotional support, psychoeducation, self-reflection, and preliminary psychological support. Unless a specific technology is being discussed, this study uses AI-based mental health services to refer to the broader service category examined in the survey. Compared with traditional face-to-face psychological counseling, AI-based mental health tools may offer advantages such as rapid response, relatively low barriers to use, and fewer restrictions related to time and space. Existing studies have evaluated such tools primarily as forms of digital self-help, psychoeducation, emotional support, or adjunctive support, rather than as replacements for professional psychological counseling (Fitzpatrick et al., 2017; Vaidyam et al., 2019). Researchers have found that users show a high level of engagement when using generative AI chatbots for mental health support and report positive experiences in emotional expression, relationship repair, and trauma coping (Siddals et al., 2024). However, despite their growing availability, students’ actual acceptance of AI-based mental health support remains uncertain.

Within technology-acceptance research, perceived usefulness and facilitating conditions or accessibility have been examined as important antecedents of perceived benefits and behavioral intention (Naji et al., 2025). However, when general-purpose large language model chatbots are used in mental health contexts, users may mistake the human-like empathy displayed by AI for genuine responsibility and may underestimate the privacy risks associated with emotional and psychological disclosure (Kwesi et al., 2025). Trust has also been repeatedly identified as an important factor in users’ acceptance of conversational AI and AI-enabled healthcare technologies, while privacy concerns may reduce willingness to adopt such systems, particularly in mental-health-related contexts (Leschanowsky et al., 2024; Kelly et al., 2025; Fan et al., 2026). In parallel, studies of conversational AI have shown that anthropomorphic cues can influence initial trust and other acceptance-related evaluations (Sfar et al., 2025), while empathy and supportive interaction have increasingly been discussed as relevant relational characteristics in users’ evaluations of mental health chatbots. However, these findings are distributed across different technological contexts, populations, and theoretical frameworks. In particular, it remains unclear how relational perceptions and privacy-related risk judgments are connected to the utility-centered mechanism of the Technology Acceptance Model (TAM), and whether trust is associated with the connections among these different evaluations to perceived usefulness and behavioral intention. Therefore, based on an extended TAM, it is necessary to further examine the pattern of factors associated with college students’ intention to use AI-based mental health services.

College students represent a population with substantial and diverse mental health needs. Large-scale cross-national evidence from the WHO World Mental Health International College Student Project has documented a considerable prevalence of common mental health problems among university students, while a substantial proportion of students with mental health problems do not receive professional treatment (Auerbach et al., 2018; Bruffaerts et al., 2019). Moreover, systematic reviews have identified a persistent gap between students’ mental health needs and professional help-seeking, with barriers including self-reliance, stigma, time constraints, limited knowledge of available resources, and low perceived need for professional support (Lui et al., 2024; Zhao et al., 2025). Against this background, AI-based mental health services may provide an additional channel through which some college students engage in self-expression, psychoeducation, and preliminary support during periods of emotional distress. However, the acceptance of AI-based mental health services cannot be simply understood as a general issue of information technology use. Mental health service contexts are highly sensitive, and users often need to disclose private information such as personal emotions, psychological distress, interpersonal relationships, and even traumatic experiences during use. Therefore, whether users are willing to use AI-based mental health services depends not only on whether they perceive the tool as useful, but also on whether they trust the system, worry about the misuse of personal information, perceive the responses as understanding and supportive, and can access the service conveniently (Davis, 1989; Gefen et al., 2003; Iwaya et al., 2023).

Based on the above, this study focuses on college students and constructs an extended Technology Acceptance Model centered on behavioral intention to use AI-based mental health services. Taking trust as the core variable, this study introduces factors such as perceived anthropomorphism, perceived empathy, perceived usefulness, privacy concern, and accessibility, aiming to explain how college students form behavioral intentions to use AI-based mental health services in the highly sensitive context of mental health services. This study seeks to examine the pattern of associations underlying intention to use AI-based mental health services and to provide empirical evidence for the design, application, and risk governance of digital mental health services in universities.

## 2. Literature Review and Research Hypotheses

### 2.1. AI-Based Mental Health Services in University Mental Health Support

AI-based mental health services encompass several technologically and functionally distinct forms of conversational support. One category consists of mental-health-specific conversational agents, such as Woebot and Wysa, which are explicitly designed to deliver structured psychological support, including psychoeducation, cognitive behavioral therapy (CBT)-based self-help, emotion-regulation exercises, symptom monitoring, and referral or signposting when additional support is needed. For example, Woebot has been evaluated among young adults and university populations as a conversational system delivering CBT-based self-help rather than as a replacement for professional psychotherapy (Fitzpatrick et al., 2017). Another category involves general-purpose generative AI platforms based on large language models. Although these systems are not primarily designed as mental health interventions, users may employ them for emotional expression, self-reflection, mental health information seeking, coping suggestions, and informal emotional support.

In the present study, the term “AI-based mental health services” is used in a broad, usage-oriented sense to refer to conversational AI tools that college students may use for mental-health-related purposes. These purposes primarily include psychoeducation, self-help, emotional support, self-reflection, and preliminary psychological support. The term does not imply that such systems provide formal diagnosis, psychotherapy, or other clinician-delivered interventions, unless the system has been specifically developed and evaluated for such purposes. In university settings, these tools are increasingly positioned as low-threshold or supplementary resources rather than substitutes for campus counseling services. Their roles may include providing immediate emotional support, self-guided psychological exercises, psychoeducational information, early-stage support, and referral to formal services when necessary. For example, Wysa has been implemented within a university support system as a scalable digital mental health resource (Khan et al., 2025), with users accessing conversational support while higher-risk cases can be directed toward appropriate professional or emergency services.

In recent years, AI-based mental health services among college students have also attracted increasing attention. Based on an open trial study, Reyes-Portillo et al. (2025) found that generative AI-driven mental health chatbots can integrate evidence-based approaches such as cognitive behavioral therapy, dialectical behavior therapy, acceptance and commitment therapy, and mindfulness interventions to provide structured and personalized conversational psychological support for college students. Their findings also showed that such tools were associated with reductions in anxiety, depression, and hopelessness in short-term follow-up, as well as improvements in self-efficacy and subjective well-being.

Although AI-based mental health services have the potential to supplement university mental health support resources, their application still faces clear limitations. Rackoff et al. (2025) found that nearly half of college students had used chatbots for various purposes, but only a small proportion had used chatbots to seek mental health support, and their attitudes toward chatbot-based psychological support were significantly less positive than their attitudes toward traditional psychological services. Cho et al. (2025) pointed out that college students’ use of digital mental health services is still influenced by factors such as resource accessibility, time constraints, financial pressure, and content clarity. Existing studies have also highlighted privacy, security, and trust as important concerns shaping users’ acceptance of conversational AI in mental health contexts (Leschanowsky et al., 2024). Mayor (2025) found that some users felt that chatbots lacked empathy and produced repetitive responses during interactions.

Taken together, previous research points to three broad domains of factors relevant to the acceptance of AI-based mental health services. First, functional evaluations, including accessibility and perceived usefulness, concern whether users regard AI-based support as available and practically valuable (Naji et al., 2025; Pitts & Motamedi, 2026). Second, relational perceptions, including anthropomorphism and perceived empathy, concern whether users experience AI interaction as human-like, understanding, and supportive (Ma et al., 2025; Niu et al., 2026). Third, risk-related evaluations, particularly privacy concern and trust, reflect users’ judgments about the vulnerability and uncertainty involved in disclosing sensitive information to conversational AI (Leschanowsky et al., 2024; Iwaya et al., 2023). Recent systematic-review evidence likewise identifies accessibility, non-judgmental interaction, privacy concerns, and safety-related uncertainty as recurring facilitators and barriers to the use of LLM-based conversational agents in mental healthcare (Shankar et al., 2026). Although prior studies provide evidence for individual relationships within these domains, less attention has been paid to how the three domains operate together in shaping college students’ acceptance of AI-based mental health services.

### 2.2. Theoretical Rationale for Extending the Technology Acceptance Model

The original TAM explains technology acceptance primarily through users’ evaluations of technological usefulness and ease of use (Davis, 1989). However, mental-health-related AI use involves not only instrumental evaluation but also access to support, interpersonal-like interaction, and the disclosure of sensitive psychological information. Accordingly, the present study extends TAM across three theoretically relevant domains: functional access, represented by accessibility; relational evaluation, represented by perceived anthropomorphism, perceived empathy, and trust; and privacy-related risk evaluation, represented by privacy concern and trust. Perceived usefulness is retained as a core TAM construct linking these context-specific evaluations to behavioral intention. The conceptual model distinguishes among exogenous constructs, mediating endogenous constructs, and the final endogenous outcome. Accessibility (ACC), Perceived Anthropomorphism (PA), and Privacy Concern (PC) are specified as exogenous constructs. Perceived Empathy (EMP), Trust (TRU), and Perceived Usefulness (PU) are endogenous constructs that also serve as mediators in the theoretically specified pathways, while Behavioral Intention (BI) is the final endogenous outcome. PEOU was not replaced by accessibility in the present model. Rather, the study retained perceived usefulness as the focal core TAM construct and introduced accessibility as a context-specific external variable because the research question concerns access to AI-based mental health support rather than interface usability. This specification is also consistent with extended TAM research in which external variables are theorized as antecedents of core acceptance beliefs rather than substitutes for them (Venkatesh & Bala, 2008).

Other factors, including AI literacy, prior experience, self-efficacy, social influence, and AI-related anxiety, may also contribute to AI acceptance and have been examined in extended technology-acceptance research. For example, recent studies have incorporated AI literacy into models of university students’ adoption of generative AI, while prior experience has also been considered as an antecedent or moderator of technology acceptance (X. Zhang et al., 2025). The present study does not regard these factors as theoretically unimportant. Rather, its scope is deliberately focused on evaluations arising from the service-use context—whether AI-based mental health support is accessible, relationally responsive, useful, trustworthy, and sufficiently safe for sensitive disclosure—rather than on users’ pre-existing competencies or experience with AI.

### 2.3. Accessibility, Perceived Usefulness, and Behavioral Intention

In the present study, accessibility is conceptualized as a perceived construct, referring to individuals’ subjective evaluation of how easily AI-based mental health services can be accessed when needed, with respect to factors such as time, space, cost, and resource availability (Levesque et al., 2013). Accessibility and perceived ease of use refer to different aspects of technology acceptance. In the original TAM, perceived ease of use refers to the extent to which using a technology is perceived as requiring little effort (Davis, 1989). By contrast, accessibility in the present study concerns whether the service can be reached or obtained when support is needed, rather than how cognitively or operationally easy the interface is to use. Thus, a service may be highly accessible—for example, available at any time and without geographical restrictions—while still being perceived as difficult to use. Conversely, an easy-to-use system may remain inaccessible because of cost, availability, or other access barriers. College students face multidimensional psychological challenges during their growth and development, including academic pressure, interpersonal adjustment, career uncertainty, and changes in self-perception. These challenges increase their demand for psychological support that is both timely and privacy-preserving. However, traditional university counseling services are often constrained by limited counseling resources, long appointment waiting times, fixed service hours, and stigma related to help-seeking. As a result, some students may not be able to seek formal help in a timely manner even when they experience psychological distress (Locke et al., 2024; Rackoff et al., 2025).

Compared with traditional face-to-face counseling, AI-based mental health tools usually have characteristics such as round-the-clock response, stronger anonymity, lower cost, and lower operational barriers. In the digital mental health research by Palmer and Schwan (2025), accessibility is also commonly regarded as a factor that can, to some extent, compensate for the limitations of offline psychological services. AlMaskari et al.’s (2025) qualitative study on AI mental health chatbots among college students also showed that many students were willing to use such tools, and an important reason was their immediacy, availability, and relatively low psychological pressure. Therefore, the following hypothesis is proposed:

**H1.**  *Accessibility is positively associated with college students’ behavioral intention to use AI-based mental health services.*

Accessibility may also influence college students’ evaluation of the usefulness of AI-based mental health services. Perceived usefulness is a core variable in TAM for explaining behavioral intention, referring to the extent to which users believe that a technology can help them improve task performance or solve practical problems (Davis, 1989). Naji et al.’s (2025) study on the adoption of mental health chatbots also indicated that facilitating conditions, accessibility, and technical support affect users’ perception of the usefulness and value of mental health chatbots. Therefore, in the context of college students’ use of AI-based mental health services, accessibility may enable users to obtain emotional support and psychological adjustment resources more quickly, thereby enhancing their perceived value of such services. Therefore, the following hypothesis is proposed:

**H2a.**  *Accessibility is positively associated with college students’ perceived usefulness of AI-based mental health services.*

Pitts and Motamedi’s (2026) study showed that perceived usefulness remains an important factor in predicting students’ intention to use AI chatbots. Therefore, for college students, if AI-based mental health tools can provide timely feedback, emotional comfort, stress management suggestions, mental health knowledge, or self-regulation guidance, they are more likely to perceive such tools as practically valuable and further develop an intention to use them. Therefore, the following hypothesis is proposed:

**H2b.**  *Perceived usefulness is positively associated with college students’ behavioral intention to use AI-based mental health services.*

**H2.**  *Accessibility is positively and indirectly associated with college students’ behavioral intention to use AI-based mental health services through perceived usefulness.*

### 2.4. Perceived Anthropomorphism, Perceived Empathy, and Trust

Perceived anthropomorphism refers to the extent to which users perceive an AI system as displaying human-like characteristics in its communication and interaction, such as naturalness of expression and the experience of interacting with a human-like social agent (Epley et al., 2007). Conceptually, anthropomorphism concerns perceived human-likeness rather than whether the system genuinely possesses emotions or empathic capacity.

Perceived empathy, by contrast, concerns users’ perception of the communicative qualities of the AI’s responses—specifically, whether its responses convey understanding, care, and appropriate responsiveness to the user’s emotions and thoughts (Niu et al., 2026). Thus, perceived empathy reflects users’ perception that the system communicates in an empathic manner. The study by Ma et al. (2025) further demonstrated that the anthropomorphic features of chatbots can significantly enhance users’ perceived empathy levels. This indicates that users’ judgment of AI’s emotional capabilities is not only based on functional performance but is also influenced by its human-like cues. Hence, perceived anthropomorphism should be regarded as a key antecedent of perceived empathy. Accordingly, the following hypothesis is proposed:

**H3a.**  *Perceived Anthropomorphism is positively associated with college students’ perceived empathy toward AI-based mental health services.*

Trust is conceptually distinct from both constructs because it concerns users’ evaluative belief that the AI system is credible, reliable, and dependable, rather than whether it appears human-like or communicates empathically (Mayer et al., 1995; McKnight et al., 2002). Accordingly, an AI system may be perceived as human-like without necessarily being regarded as empathic or trustworthy; similarly, an empathically worded response does not necessarily imply that the user considers the system reliable or safe to depend on. Niu et al. (2026) found in their study on generative AI and information disclosure that perceived empathy can positively influence users’ trust in generative AI and further affect their disclosure intention. Deng and Yan’s (2025) study also showed that social attributes such as chatbot warmth and social presence can influence users’ continued usage intention through trust. In the context of AI-based mental health services for college students, when users believe that AI can understand their emotions and provide appropriate responses, they are more likely to believe that the system is supportive, reliable, and helpful (Ding & Barbic, 2025; Rządeczka et al., 2025). Accordingly, the following hypothesis is proposed:

**H3b.**  *Perceived empathy is positively associated with college students’ trust in AI-based mental health services.*

**H3.**  *Perceived anthropomorphism is positively and indirectly associated with college students’ trust in AI-based mental health services through perceived empathy.*

### 2.5. Privacy Concern, Trust, and Behavioral Intention

Privacy concern has been identified as a potential barrier to adoption of AI-based mental health services. Privacy concern generally refers to users’ worries about the collection, storage, use, sharing, or misuse of their personal information (Malhotra et al., 2004). Privacy concern may reduce users’ intention to use AI-based mental health services. Lawati et al. (2026), in their study on undergraduate students’ attitudes toward AI mental health chatbots, showed that although college students generally possess relatively high digital literacy, they still have concerns about data security and regulation regarding AI-based mental health tools. These concerns may limit trust, comfort, and acceptance. Naji et al. (2025) also pointed out that in the adoption of mental health chatbots, trust, privacy, and credibility may influence users’ decision-making more strongly than perceived value alone. Therefore, when users worry that their emotional states, psychological distress, or personal privacy data may be collected, leaked, or misused, their trust in and willingness to use AI-based mental health services may decrease. Accordingly, the following hypothesis is proposed:

**H4.**  *Privacy concern is negatively associated with college students’ behavioral intention to use AI-based mental health services.*

In recent years, studies on conversational AI and mental health applications have emphasized the close relationship between privacy and trust. Leschanowsky et al. (2024), in a systematic review of conversational AI, pointed out that privacy, security, and trust are not independent factors, but are intertwined in the process of users’ acceptance and use of conversational AI. Kauttonen et al.’s (2025) survey on the acceptance of AI in healthcare also showed that users’ trust in and acceptance of AI health applications are influenced by factors such as risk perception. Iwaya et al. (2023) further noted that mental health applications have problems regarding data collection and the transparency of privacy policies, which may intensify users’ concerns about digital mental health services.

In the context of generative AI-based mental health services, privacy concerns may be further intensified due to the openness and uncertainty of AI. Kwesi et al. (2025) pointed out that when users communicate with general-purpose large language model chatbots about mental health-related issues, they may underestimate the privacy risks associated with psychological disclosure and may mistake the human-like empathy displayed by AI for genuine responsibility. This suggests that the more naturally and intimately AI interacts with users, the more likely users may be to disclose sensitive information. However, such disclosure does not necessarily mean that users truly understand the risks associated with their data. Therefore, when college students worry that their personal psychological information may be improperly collected, analyzed, or used for purposes without their consent, their trust in AI-based mental health services is likely to decline. Based on this, the following hypothesis is proposed:

**H5a.**  *Privacy concern is negatively associated with college students’ trust in AI-based mental health services.*

Furthermore, previous studies have shown that trust can reduce users’ concerns when they encounter technological uncertainty and thereby facilitate technology adoption (Gefen et al., 2003). Guo et al. (2025) also demonstrated that trust is an important determinant of users’ acceptance of AI-generated health information. In the context of AI-based mental health services, college students are more likely to use such tools when experiencing psychological distress if they perceive them as reliable and safe, believe that their responses are accurate, and recognize their potential supportive value. Accordingly, the following hypothesis is proposed:

**H5b.**  *Trust is positively associated with college students’ behavioral intention to use AI-based mental health services.*

**H5.**  *Privacy concern is negatively and indirectly associated with college students’ behavioral intention to use AI-based mental health services through trust.*

Trust is a multidimensional concept that has been theorized in terms of dimensions such as ability, benevolence, and integrity (Mayer et al., 1995), while technology-related trust research has also emphasized users’ beliefs regarding the reliability and trustworthiness of technological systems (McKnight et al., 2002; Gefen et al., 2003). Given the high levels of uncertainty and risk associated with mental health services, trust in AI is particularly important in this context compared with more general technology-use settings. Trust may also shape users’ perceptions of the usefulness of AI-based mental health services. Perceived usefulness is not merely an evaluation of a system’s functionality; it is also influenced by users’ judgments regarding the reliability of the information source. When users do not trust the advice provided by AI, they may regard the information as lacking practical value, even when the system offers substantial psychological knowledge and emotion-regulation strategies. Conversely, when users perceive the information and suggestions provided by an AI system as reliable, accurate, and credible, they are more likely to regard its outputs as useful resources for psychological support. Gefen et al. (2003) argued that trust not only directly influences behavioral intention but may also shape users’ evaluations of technological value. Similarly, Pitts and Motamedi (2026) found that trust affects students’ perceptions of the usefulness of conversational AI. Therefore, in the context of AI-based mental health services, trust may further promote behavioral intention by enhancing perceived usefulness. Accordingly, the following hypothesis is proposed:

**H6.**  *Trust is positively associated with college students’ perceived usefulness of AI-based mental health services.*

In the present model, privacy concern is treated as an exogenous predictor rather than as an outcome of perceived anthropomorphism or perceived empathy. However, this modeling choice does not imply that privacy concern is necessarily independent of users’ relational experiences with AI. Previous research suggests that anthropomorphic and socially engaging characteristics of conversational agents may influence users’ privacy concerns and self-disclosure decisions (Lee & Lee, 2023; M. Zhang & Zhu, 2026). In mental health-related interactions, human-like and emotionally supportive features may also facilitate users’ willingness to disclose personal information (Lee & Lee, 2023). At the same time, the relationship between anthropomorphic interaction and privacy-related perceptions may be context-dependent rather than uniformly directional, as anthropomorphic cues can alter both the perceived benefits and privacy risks associated with disclosure (M. Zhang & Zhu, 2026; Nikolov et al., 2026). Conversely, heightened privacy concern may influence users’ willingness to disclose sensitive information and their broader evaluations of AI-based services (Leschanowsky et al., 2024; M. Zhang & Zhu, 2026). These possible reciprocal or dynamic relationships fall outside the scope of the present cross-sectional model.

**Figure 1:**
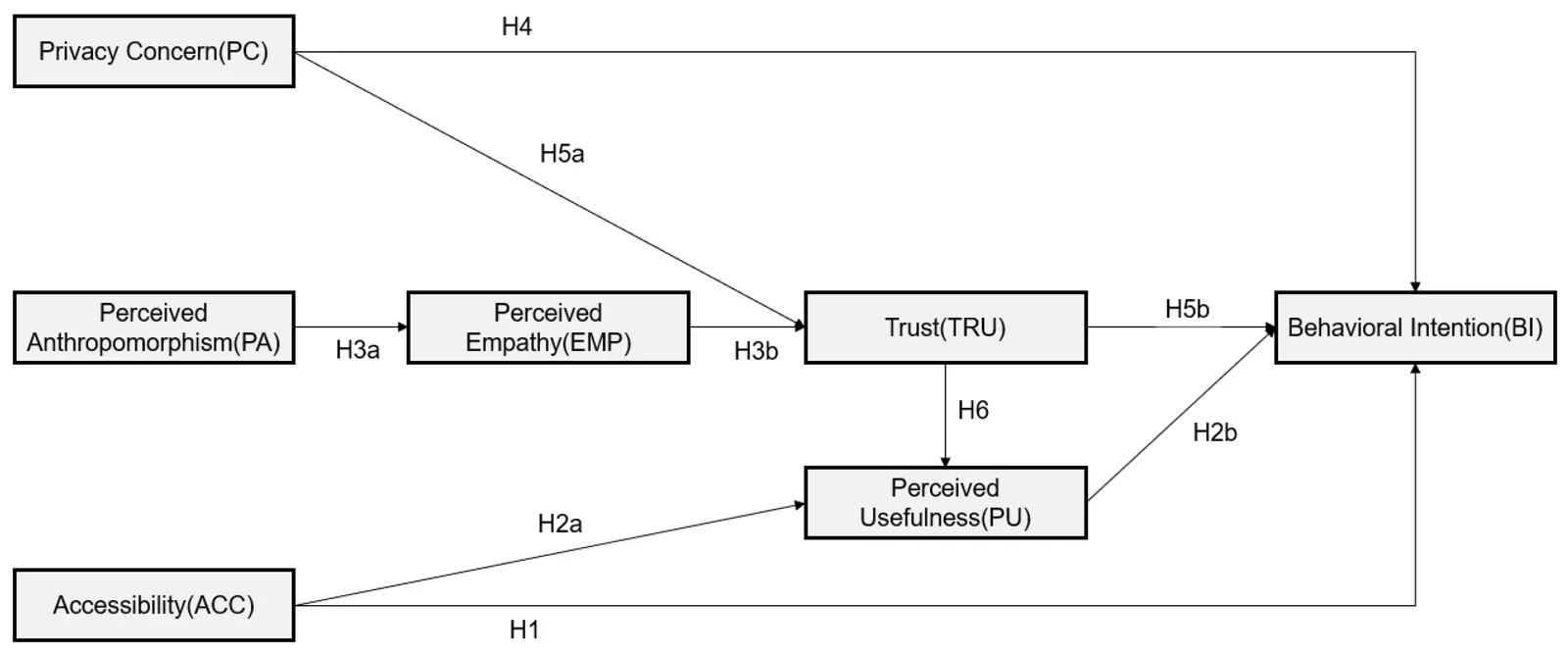
Research hypotheses.

In summary, we present the hypothesized framework of this study, as illustrated in Figure 1.

## 3. Research Methodology

### 3.1. Participants

This study employed convenience sampling to recruit college students from multiple higher education institutions across China. The initial questionnaire link was distributed by the researchers to college students within their personal networks and was subsequently circulated through WeChat group chats and Moments. No specific university, course, faculty, major, or degree program was deliberately targeted. Eligibility was based solely on current college student status. At the beginning of the questionnaire, respondents were asked to confirm that they were currently enrolled college students; those who did not meet this criterion exited the survey. No additional eligibility requirements, such as prior experience with psychological counseling or AI-based mental health services, were imposed.

A total of 383 questionnaires were submitted, of which 375 were retained, and the valid questionnaire rate was 97.91%. The adequacy of the sample size was evaluated using the inverse square root method (Kock & Hadaya, 2018). At a 5% significance level and 80% statistical power, a sample of 375 allows path coefficients with an absolute value of approximately 0.13 or larger to be detected reliably. Most hypothesized paths exceeded this value. Among the final sample, 222 participants were female (59.2%) and 153 were male (40.8%). And 60.53% of the respondents reported no prior experience with psychological counseling. Age and field of study were not collected in the survey. All participants provided written informed consent, and this study was approved by the Departmental Ethics Committee and the Institutional Review Board of Guangdong University of Technology (Approval No. GDUTXS20260198). Table 1 presents the demographic information of the participants.

### 3.2. Development of Measurement Instruments

A questionnaire developed through a series of steps was used to measure the research variables. First, relevant literature on the use of AI chatbots was reviewed, and some items were adjusted according to the theme of this study, namely the use of AI chatbots for mental health purposes. It should be noted that the original questionnaire contained some variation in the terminology used across measurement items, including references to “generative artificial intelligence”, “AI psychological counseling tools,” and “AI-based mental health services.” These expressions were retained from the adapted measurement items and were presented to participants without specifying a particular AI platform or technological architecture. Therefore, these tools could include either dedicated mental health chatbots or general-purpose generative AI systems when used for such purposes. The term did not refer exclusively to formal clinical psychotherapy or diagnosis. Subsequently, three experts with relevant academic backgrounds were invited to review the questionnaire. The expert panel consisted of a professor and doctoral supervisor specializing in applied psychology and behavioral science, an associate professor and doctoral supervisor specializing in human–computer interaction and user behavior, and an associate professor and master’s supervisor with expertise in research methodology, psychometric measurement, and questionnaire design. The experts independently evaluated the questionnaire in terms of item clarity, content relevance, construct consistency, and wording appropriateness. Based on their feedback, several items were refined to improve clarity and ensure consistency with the conceptual definitions of the constructs. The final questionnaire consisted of two parts. The first part collected demographic information, including gender, grade level, and previous experience with psychological counseling. The second part assessed the seven variables included in the research model.

The questionnaire was administered in Chinese. Before the formal survey, a pilot test was conducted with 66 college students who were not included in the final analytical sample. The pilot test was used to evaluate the clarity of item wording, the overall questionnaire procedure, completion time, and the preliminary reliability of the adapted measures. During the development of the questionnaire, no formal cognitive interviews were conducted and no formal reverse translation process was carried out. The complete list of items for each scale and their reference sources can be found in Appendix A.

#### 3.2.1. Accessibility Scale

Based on the Accessibility Scale developed by Wu and Wang (2025), respondents were asked to evaluate their perceived accessibility of AI-based mental health services, that is, the extent to which they believed such services could be conveniently accessed when needed. For example, “I can use AI-based mental health services without restrictions of time and place.” A five-point Likert scale was adopted, and the scale consisted of three items. In this study, the Cronbach’s alpha coefficient of this scale was 0.788.

#### 3.2.2. Privacy Concern Scale

Based on the Privacy Concern Scale developed by Niu et al. (2026), respondents were asked to evaluate their concerns about personal information security and privacy protection in AI-based mental health services. For example, “I am concerned that the personal information I provide to AI may be used for purposes to which I have not consented”. A five-point Likert scale was adopted, and the scale consisted of four items. In this study, the Cronbach’s alpha coefficient of this scale was 0.855.

#### 3.2.3. Perceived Empathy Scale

Based on the Perceived Empathy Scale developed by Niu et al. (2026), respondents were asked to evaluate their perception of whether the responses generated by AI conveyed understanding, care, and appropriate responsiveness to their emotions and thoughts. For example, “I believe that generative artificial intelligence can understand my feelings and thoughts”. A five-point Likert scale was adopted, and the scale consisted of three items. In this study, the Cronbach’s alpha coefficient of this scale was 0.828.

#### 3.2.4. Perceived Usefulness Scale

Based on the Perceived Usefulness Scale developed by Sharma et al. (2025), respondents were asked to evaluate the practical value of AI-based mental health tools in emotional regulation and psychological counseling. For example, “Using AI-based mental health tools can help me relieve negative emotions more quickly”. A five-point Likert scale was adopted, and the scale consisted of five items. In this study, the Cronbach’s alpha coefficient of this scale was 0.886.

#### 3.2.5. Perceived Anthropomorphism Scale

Based on the Perceived Anthropomorphism Scale developed by Balakrishnan et al. (2022) and Li et al. (2025), respondents were asked to evaluate perceived human-likeness in AI-mediated interaction, including the naturalness of responses, the sense of communicating with a human-like social agent, and the interpersonal atmosphere experienced during interaction. For example, “The responses from AI are natural, and I do not feel that they are artificial”. A five-point Likert scale was adopted, and the scale consisted of three items. In this study, the Cronbach’s alpha coefficient of this scale was 0.817.

#### 3.2.6. Trust Scale

Based on the Trust Scale developed by Niu et al. (2026), respondents were asked to evaluate the credibility, information accuracy, and reliability of suggestions provided by generative AI-based mental health services. For example, “I believe that generative artificial intelligence is trustworthy”. A five-point Likert scale was adopted, and the scale consisted of five items. In this study, the Cronbach’s alpha coefficient of this scale was 0.879.

#### 3.2.7. Behavioral Intention Scale

Based on the Behavioral Intention Scale developed by Wijaya et al. (2025), respondents were asked to evaluate their behavioral tendency to use, continue using, and recommend AI psychological counseling tools in the future. For example, “I intend to use AI psychological counseling tools in the future to cope with my psychological distress”. A five-point Likert scale was adopted, and the scale consisted of five items. In this study, the Cronbach’s alpha coefficient of this scale was 0.854.

### 3.3. Data Collection

Data were collected from 20 April to 23 April 2026, using Wenjuanxing (Changsha, China), an online questionnaire platform. The questionnaire link was initially distributed by the researchers to college students within their personal networks and was subsequently shared through WeChat group chats and Moments. Participation was voluntary, and respondents who completed the questionnaire received a CNY 1 monetary incentive. To ensure data quality, responses were screened before data analysis. Four questionnaires with completion times of less than 65 s were excluded. Two questionnaires were excluded because they showed patterned responding across the Likert-scale items. An additional two questionnaires were excluded because they were submitted from duplicate IP addresses. Thus, 8 of the 383 submitted questionnaires were excluded, leaving 375 valid responses for analysis. All questionnaire items were set as mandatory in the online survey; therefore, there were no missing data in the submitted questionnaires, and no missing-data imputation was performed. Subsequently, reliability and validity tests were conducted on the valid samples to ensure the reliability of the subsequent data analysis results.

The questionnaire did not specify a particular AI-based mental health platform or provide a standardized interaction scenario or demonstration. Participants responded to the items based on their general understanding of AI-based mental health services. Accordingly, the accessibility measure should be interpreted as perceived or anticipated accessibility rather than as an evaluation derived necessarily from direct use experience. Respondents without prior experience with AI-based mental health services could form such evaluations based on their general knowledge, expectations, or indirect exposure to AI services. Moreover, behavioral intention in this study should be interpreted primarily as participants’ prospective intention to use a broadly defined or hypothetical AI-based mental health service, rather than as post-use acceptance based on direct experience with a standardized AI mental health system. Because the original questionnaire used somewhat different technology-related expressions across several adapted items, participants may not necessarily have interpreted all items as referring to exactly the same technological form. This issue should be considered when interpreting the focal construct and the resulting associations.

Common method variance (CMV) was addressed procedurally by guaranteeing anonymity and statistically using Harman’s single-factor test (Podsakoff et al., 2003). In Harman’s single-factor test on the 28 Likert-type items, the first unrotated factor explained 35.69% of the variance, below the 50% heuristic.

## 4. Results

### 4.1. Reliability and Validity Analysis

In this study, SmartPLS (version 4.1.1.6; SmartPLS GmbH, Monheim am Rhein, Germany) was used to assess the measurement model, including indicator loadings, internal consistency reliability, convergent validity, and discriminant validity. Reliability analysis was conducted on the sample, and the Cronbach’s alpha values of all constructs were greater than 0.7, indicating that the collected data showed good internal consistency and strong reliability.

Subsequently, the measurement model was evaluated in SmartPLS using outer loadings, Cronbach’s α, composite reliability (CR), and average variance extracted (AVE). CR was used to assess whether the items consistently reflected their corresponding constructs, while AVE was used to indicate the extent to which a construct could explain the variance of its measurement items. In this study, the CR values of all variables exceeded 0.7, and all AVE values were greater than 0.5 (Fornell & Larcker, 1981), meeting the criteria for convergent validity. Therefore, the overall convergent validity reached an acceptable standard. The results of reliability and convergent validity are shown in Table 2.

Finally, discriminant validity was examined. The values on the diagonal of the table represent the square roots of the AVE values calculated using SmartPLS. In validity analysis, discriminant validity requires that the square root of the AVE value for each variable be greater than the correlation coefficients between that variable and other variables. Table 3 presents the results of the discriminant validity test for the sample. The correlation coefficients among the variables were smaller than the square roots of the AVE values on the diagonal, indicating that the questionnaire had acceptable discriminant validity (Fornell & Larcker, 1981).

In addition to the Fornell–Larcker criterion, discriminant validity was further assessed using the heterotrait–monotrait ratio of correlations (HTMT), as recommended by Henseler et al. (2015). HTMT values below 0.85 indicate adequate discriminant validity under a conservative criterion, while a threshold of 0.90 may be considered for constructs that are conceptually similar. Given the conceptual proximity among several constructs in the present model, particular attention was paid to the HTMT values for EMP–TRU and PA–EMP. As shown in Table 4, all HTMT values were below 0.85, indicating adequate discriminant validity among the constructs. Table 5 presents the descriptive statistics and correlations among the study variables.

### 4.2. Model Testing

Explanatory power was assessed with R^2^. R^2^ values of 0.25, 0.50, and 0.75 are commonly interpreted as weak, moderate, and substantial, respectively (Hair et al., 2019). As shown in Table 6, R^2^ was 0.660 for BI, 0.551 for TRU, 0.464 for PU, and 0.350 for EMP, indicating weak to moderate explanatory power. The SRMR values were 0.049 for the saturated model and 0.063 for the estimated model. The SRMR of the estimated model was below the commonly recommended threshold of 0.08, suggesting an acceptable approximate model fit (Hu & Bentler, 1999). The structural model and hypothesized paths were then assessed using SmartPLS within the PLS-SEM framework. The direct and indirect components of H2, H3, and H5 were evaluated separately. Statistical significance of the path coefficients was assessed using bootstrapping with 5000 resamples.

As shown in Table 6, all inner VIF values ranged from 1.000 to 2.002, indicating no critical collinearity concerns among the predictor constructs.

All path coefficients and indirect-effect estimates reported below are standardized estimates. The path analysis results indicate that H2a and H2b are significant at the 0.05 level, H4 is significant at the 0.01 level, while H1, H3a, H3b, H5a, H5b, and H6 are all significant at the 0.001 level. Table 7 summarizes the results of all hypothesis tests.

The mediation effects were further examined in SmartPLS using bootstrapping with 5000 resamples and 95% confidence intervals. The results are presented in Table 8. The results of the mediation effect analysis showed that all seven specific indirect effects had confidence intervals that did not include zero, indicating that the mediation effects were significant. For H2, the specific indirect effect of ACC on BI through PU was very small (β = 0.012). The 95% percentile bootstrap confidence interval excluded zero (95% CI [0.001, 0.027]), whereas the corresponding t-statistic approximation yielded *p* = 0.085. Thus, the statistical conclusion for this indirect effect was sensitive to the inferential criterion applied. Given the very small effect magnitude and the proximity of the lower bootstrap confidence bound to zero, this finding should be interpreted cautiously. For H3, the indirect effect of PA on TRU through EMP was statistically significant (β = 0.416, 95% CI [0.353, 0.476]), supporting H3. For H5, the indirect effect of PC on BI through TRU was also statistically significant (β = −0.126, 95% CI [−0.173, −0.085]), supporting H5. The corresponding direct paths were also statistically significant. Additional specific indirect effects are reported in Table 8 using their full path descriptions.

**Figure 2:**
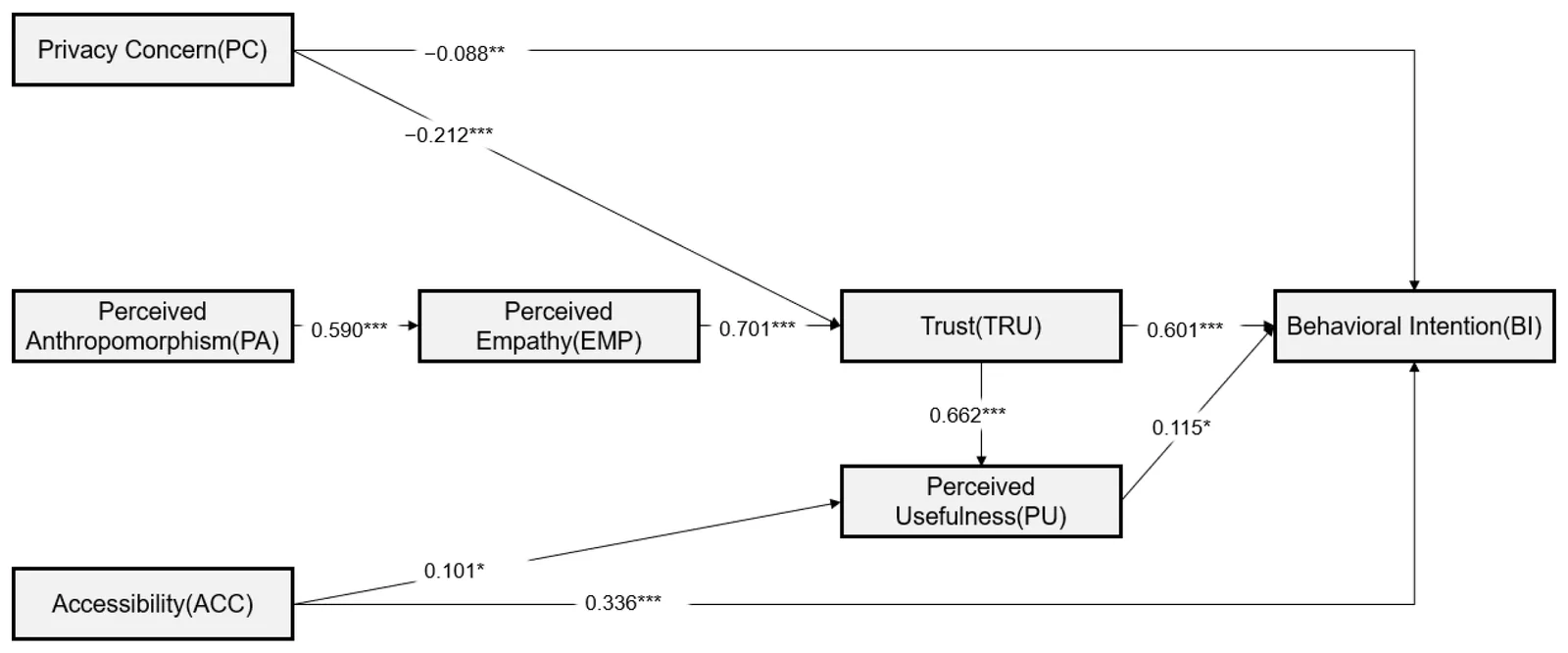
Structural model, * *p* < 0.05, ** *p* < 0.01, *** *p* < 0.001.

The final structural model and the standardized path coefficients are presented in Figure 2.

## 5. Discussion

This study examined college students’ intention to use AI-based mental health services through an extended Technology Acceptance Model incorporating accessibility, privacy concern, perceived anthropomorphism, perceived empathy, trust, and perceived usefulness. The findings suggest that technology acceptance in this context involves considerations beyond the utility-centered evaluations traditionally emphasized in TAM. In particular, trust emerged as a central construct associated with relational evaluations, privacy-related risk perceptions, and the acceptance of AI-based mental health services. This pattern is especially relevant to mental health services, where technology use involves not only judgments about functional value but also decisions about whether a system can be relied upon when users disclose sensitive psychological information.

### 5.1. Research Findings

First, trust emerged as a central factor associated with college students’ behavioral intention to use AI-based mental health services. Trust was positively associated with both behavioral intention and perceived usefulness, while perceived usefulness was also positively associated with behavioral intention. These findings extend the utility-centered logic of TAM by suggesting that acceptance in this context involves not only evaluations of functional value but also judgments about whether AI can be relied upon for psychological support. This interpretation is consistent with trust theory, which emphasizes the role of trust in reducing uncertainty and facilitating behavioral intention under conditions of risk and information asymmetry (Gefen et al., 2003; McKnight et al., 2002).

Second, privacy concern was negatively associated with both trust and behavioral intention, underscoring the relevance of privacy-related risk evaluations in the acceptance of AI-based mental health services. This pattern is consistent with previous research identifying privacy risks related to data collection, sharing, and transparency in mental health applications (Iwaya et al., 2023; Borghouts et al., 2021), as well as research linking privacy and trust in users’ evaluations of generative AI systems (Niu et al., 2026). More broadly, these findings are compatible with a privacy-calculus perspective, in which expected benefits are weighed against perceived privacy risks (von Kalckreuth & Feufel, 2023). However, privacy concern may also partly reflect broader risk sensitivity or general skepticism toward AI-based services rather than a privacy-specific process alone. Because individual differences such as risk tolerance and prior AI experience were not assessed in the present study, this possibility cannot be ruled out. Therefore, the observed associations should be interpreted as evidence of the relevance of privacy-related evaluations to trust and behavioral intention, rather than as evidence that privacy concern causally reduces trust or acceptance.

Third, the relevance of accessibility suggests that the appeal of AI-based mental health services may not be limited to their perceived functional usefulness. Features such as flexible access, low thresholds for engagement, and reduced temporal or spatial constraints may be particularly relevant to college students who face practical or psychological barriers to conventional help-seeking. This interpretation is broadly consistent with previous research highlighting time constraints, limited resources, and help-seeking stigma as barriers to conventional mental health support (Broglia et al., 2021; Palmer & Schwan, 2025). Nevertheless, these contextual factors were not directly measured in the present study. Therefore, the observed association with accessibility should not be taken as evidence that resource shortages or help-seeking stigma themselves account for students’ intention to use AI-based services.

Taken together, the pattern of indirect associations suggests that students’ acceptance of AI-based mental health services is related to multiple types of evaluation rather than to a single instrumental judgment. Relational perceptions, including anthropomorphism and perceived empathy, appear to be closely connected with trust, whereas privacy-related concerns are associated with less favorable trust evaluations. Trust, in turn, is linked with both perceived usefulness and behavioral intention. These findings therefore suggest that functional, relational, and risk-related evaluations may coexist within students’ overall assessments of AI-based mental health services. However, the observed indirect associations should not be interpreted as evidence of a temporal or causal sequence. Because all constructs were measured concurrently, alternative ordering is possible. For example, students with generally favorable attitudes toward AI may simultaneously report greater empathy, trust, usefulness, and behavioral intention.

### 5.2. Theoretical Contributions

This study makes the following theoretical contributions. First, this study analyzes AI-based mental health services within the specific usage context of college students, thereby responding to the insufficient understanding of user acceptance patterns and associated factors in digital mental health research. Existing research on college students has often examined utilization, attitudes, intervention outcomes, or selected adoption factors, whereas fewer studies have tested an integrated acceptance framework combining functional, relational, and privacy-related evaluations. By examining the psychological factors and relationships associated with college students’ use of AI-based mental health services, this study provides a new empirical perspective for research on digital mental health services in universities.

The present model extends a primarily utility-centered account of technology acceptance by simultaneously incorporating functional, relational, and risk-related evaluations. More specifically, the model positions trust as a bridging construct linking relational perceptions and privacy-related risk evaluations with perceived usefulness and behavioral intention. In addition, this study incorporates PC as a negatively associated risk-related evaluation into the model. The findings regarding PC indicate that, in the mental health context, risk factors should not be understood merely as the opposite of functional advantages. Therefore, future research on AI-based mental health services should not only discuss how such tools can become more useful and convenient, but also pay attention to factors associated with why users may distrust them or be unwilling to disclose personal information.

### 5.3. Practical Implications

The findings of this study provide several practical implications for the design of AI-based mental health services and the construction of university mental health support systems. First, AI-based mental health services should emphasize naturalness, supportiveness, and a sense of boundaries in response design. Smith et al. (2025) pointed out that, in emotional support contexts, expressing understanding, acknowledging feelings, and further exploring individuals’ emotions are important ways to support people in distress. Siddals et al.’s (2024) study on the use of generative AI for mental health also showed that positive interactive experiences are associated with greater user engagement and may contribute to positive experiences in areas such as relational understanding and trauma coping. Therefore, AI-based mental health systems may improve users’ perceived support experience through more natural, gentle, and supportive language.

Second, while providing empathic responses, AI-based mental health services should clearly define their auxiliary boundaries and incorporate appropriate safety safeguards. Human-like and supportive responses may strengthen users’ perceived connection with AI, but such perceptions should not be equated with clinical competence or professional responsibility (Kwesi et al., 2025). More importantly, generative AI may produce inaccurate, biased, or incomplete health information, while excessive reliance on AI-generated outputs may contribute to automation bias (World Health Organization, 2024). Reviews of conversational AI in mental health care have similarly identified harmful advice, crisis management, and user dependency as important safety concerns (Hua et al., 2025; Rahsepar Meadi et al., 2025). Highly agreeable or reassuring responses may also reinforce maladaptive reassurance-seeking or validate harmful beliefs rather than appropriately redirecting users (American Psychological Association, 2025). These risks are particularly consequential in situations involving self-harm, suicidal ideation, or other acute psychological crises. Current evidence does not support treating general-purpose generative AI as a substitute for qualified mental health professionals, and its capacity for reliable crisis management remains limited (American Psychological Association, 2025; Hua et al., 2025). Therefore, AI-based mental health services should clearly communicate their limitations and incorporate appropriate referral or escalation mechanisms when crisis risks are identified. These recommendations reflect broader clinical and ethical evidence rather than practices directly evaluated in the present study.

Third, platforms and universities should regard privacy protection as a fundamental condition for the design and promotion of AI-based mental health services. Leschanowsky et al.’s (2024) systematic review showed that privacy, security, and trust perceptions intersect in the use of conversational artificial intelligence and are closely associated with technology adoption and usage. Iwaya et al. (2023) also noted that mental health applications involve data privacy issues, and that users of mental health services may be more vulnerable. Therefore, AI-based mental health service platforms should inform users in a concise and understandable manner about how their data will be collected, stored, used, shared, and deleted, and should provide optional and revocable data authorization mechanisms.

Finally, the present findings suggest that AI-based mental health services may have potential as supplementary resources within broader university mental health support systems, particularly because accessibility was positively associated with students’ behavioral intention. Existing research has also indicated that chatbot-based support may face fewer barriers related to time, cost, and help-seeking stigma than some traditional services (Rackoff et al., 2025). On this basis, functions such as psychoeducation, stress-management support, emotional self-reflection, preparation for professional help-seeking, and other forms of low-intensity support may warrant further exploration in university settings. However, the present study did not evaluate the clinical effectiveness, safety, referral performance, or service-level outcomes of integrating AI into a tiered mental health system. Therefore, positioning AI as an entry point or auxiliary component within such systems should be regarded as a potential implementation strategy requiring further empirical evaluation. Future research should examine whether, for whom, and under what conditions such integration can improve accessibility without compromising privacy, safety, professional boundaries, or continuity of care.

### 5.4. Limitations and Future Research

This study still has several limitations. First, the research sample mainly consisted of Chinese college students. Although the findings can reflect the basic attitudes of this group toward AI-based mental health services, the representativeness of the sample remains limited. Future research could further expand the sources of samples, for example, by comparing the views of groups from different age ranges on AI-based mental health services.

Second, the study did not provide participants with a standardized AI-based mental health service, platform, or interaction scenario, and the questionnaire used somewhat varied terminology across items. In addition, the survey did not assess prior use of generative AI or AI for mental-health-related purposes, familiarity with AI chatbots, current psychological distress, or field of study. Consequently, heterogeneity in participants’ technological experience and interpretation of the target service may have affected their evaluations of anthropomorphism, empathy, trust, and usefulness. Future research should define a specific AI service, use consistent terminology, provide a standardized interaction context, and assess relevant participant characteristics, particularly prior AI experience and familiarity.

Third, the cross-sectional and self-report design limits causal interpretation of the observed relationships. Although the proposed model specifies directional paths among perceived empathy, trust, perceived usefulness, and behavioral intention, the present data establish associations rather than temporal or causal effects. Reverse or reciprocal relationships are also plausible. For example, students who are already more willing to use AI-based mental health services may evaluate such services as more useful, trustworthy, or empathic. Longitudinal or experimental studies are therefore needed to examine the temporal ordering and potential reciprocal relationships among these constructs. In addition, because all focal variables were measured using the same self-report questionnaire, common-method variance cannot be completely excluded despite the procedural and statistical checks employed in this study. The self-report nature of the survey may also have introduced social desirability bias. Because the study concerns mental health support and willingness to use AI in psychologically sensitive situations, some participants may have reported attitudes or intentions that they perceived as more socially acceptable rather than fully reflecting their private evaluations. Future research could incorporate a social desirability measure or use complementary behavioral and qualitative data to examine the robustness of these self-reported intentions.

Fourth, the sampling and measurement strategies also limit the generalizability and construct interpretation of the findings. Participants were recruited through convenience sampling and social-media networks, which may have introduced selection bias and limited the representativeness of the sample. Moreover, several measurement items were adapted from scales developed in related but not identical technological or service contexts. Although reliability and validity were acceptable in the present sample, contextual adaptation may not fully capture how college students evaluate AI specifically for mental-health-related purposes. Future research should employ more diverse or probability-based samples and further validate context-specific measures of AI-based mental health service acceptance. Several adapted items also present potential content-validity concerns. Although construct-level reliability and discriminant-validity results were acceptable, these findings do not eliminate item-level conceptual ambiguity. Future research should refine these items and conduct additional content-validation procedures before administration.

Finally, the present model treated privacy concern as an exogenous predictor of trust and behavioral intention and did not examine its potential relationships with perceived anthropomorphism or perceived empathy. However, relational features of AI interaction may influence privacy-related perceptions by encouraging self-disclosure or changing users’ awareness of disclosure risks. The reverse possibility is also plausible, as users with stronger privacy concerns may interpret or respond to anthropomorphic and empathic cues differently. Given the cross-sectional design, the present study cannot establish the temporal or reciprocal relationships among these constructs. Future longitudinal or experimental studies could examine whether and how anthropomorphic and empathic interactions dynamically shape privacy concern and disclosure behavior.

## 6. Conclusions

This study examined college students’ intention to use AI-based mental health services through an extended Technology Acceptance Model incorporating accessibility, privacy concern, perceived anthropomorphism, perceived empathy, trust, and perceived usefulness. The findings indicate that students’ intention to use such services is associated not only with perceived accessibility and usefulness but also with relational and risk-related evaluations. In particular, trust was positively associated with both behavioral intention and perceived usefulness, while privacy concern was negatively associated with trust and behavioral intention. Perceived anthropomorphism was positively associated with perceived empathy, which was in turn positively associated with trust. These findings suggest that understanding the acceptance of AI-based mental health services requires consideration of functional, relational, and privacy-related factors together. However, the findings should be interpreted in light of the study’s cross-sectional design, convenience sample of Chinese college students, self-reported measures, and the broadly defined AI-based mental health service context. Future longitudinal and context-specific research is needed to examine the robustness and generalizability of these associations.

## Figures and Tables

**Table 1:** Demographic characteristics (N = 375).

	Item	Number	Percentage (%)
Gender	Male	153	40.80%
	Female	222	59.20%
Grade	Freshman	73	19.47%
	Sophomore	108	28.80%
	Junior	80	21.33%
	Senior	80	21.33%
	Graduate student and above	34	9.07%
Experience in Psychological Counseling	No experience	227	60.53%
	Experienced	148	39.47%

**Table 2:** Data concerning the indicators of confidence and convergent validity used in this research.

Constructs	Item	Loadings	Cronbach α	AVE	CR
ACC	ACC1	0.859	0.788	0.702	0.876
	ACC2	0.846			
	ACC3	0.809			
PC	PC1	0.867	0.855	0.693	0.900
	PC2	0.869			
	PC3	0.781			
	PC4	0.810			
EMP	EMP1	0.857	0.828	0.744	0.897
	EMP2	0.842			
	EMP3	0.888			
PU	PU1	0.847	0.886	0.686	0.916
	PU2	0.837			
	PU3	0.801			
	PU4	0.823			
	PU5	0.833			
PA	PA1	0.846	0.817	0.732	0.891
	PA2	0.868			
	PA3	0.852			
TRU	TRU1	0.807	0.879	0.675	0.912
	TRU2	0.835			
	TRU3	0.813			
	TRU4	0.822			
	TRU5	0.830			
BI	BI1	0.791	0.854	0.632	0.896
	BI2	0.802			
	BI3	0.771			
	BI4	0.821			
	BI5	0.788			

Note: ACC = Accessibility, PC = Privacy Concern, EMP = Perceived Empathy, PU = Perceived Usefulness, PA = Perceived Anthropomorphism, TRU = Trust, BI = Behavioral Intention.

**Table 3:** Results of the validity tests with respect to sample differentiation: Fornell–Larcker Criterion.

Construct	ACC	BI	EMP	PA	PC	PU	TRU
ACC	**0.838**						
BI	0.407	**0.795**					
EMP	0.067	0.594	**0.863**				
PA	0.087	0.45	0.591	**0.856**			
PC	0.037	−0.223	−0.047	−0.023	**0.833**		
PU	0.165	0.578	0.473	0.459	−0.038	**0.828**	
TRU	0.094	0.73	0.712	0.531	−0.242	0.673	**0.821**

Note: ACC = Accessibility, PC = Privacy Concern, EMP = Perceived Empathy, PU = Perceived Usefulness, PA = Perceived Anthropomorphism, TRU = Trust, BI = Behavioral Intention.

**Table 4:** Heterotrait–Monotrait Ratio (HTMT) of Constructs.

Constructs	ACC	BI	EMP	PA	PC	PU	TRU
ACC							
BI	0.491						
EMP	0.109	0.706					
PA	0.108	0.537	0.714				
PC	0.072	0.252	0.071	0.080			
PU	0.194	0.663	0.550	0.540	0.068		
TRU	0.117	0.842	0.833	0.626	0.271	0.762	

Note: ACC = Accessibility, PC = Privacy Concern, EMP = Perceived Empathy, PU = Perceived Usefulness, PA = Perceived Anthropomorphism, TRU = Trust, BI = Behavioral Intention.

**Table 5:** Descriptive Statistics and Correlations among Study Variables.

Variable	M	SD	Skewness	Excess Kurtosis	1	2	3	4	5	6	7
1. ACC	3.662	0.802	−0.455	−0.142	—						
2. BI	3.382	0.793	−0.261	−0.555	0.404 **	—					
3. EMP	3.433	0.841	−0.396	−0.124	0.064	0.593 **	—				
4. PA	3.260	0.869	−0.310	−0.462	0.083	0.450 **	0.589 **	—			
5. PC	3.685	0.803	−0.668	−0.058	0.045	−0.216 **	−0.041	−0.013	—		
6. PU	3.523	0.796	−0.315	−0.454	0.162 **	0.577 **	0.471 **	0.459 **	−0.030	—	
7. TRU	3.379	0.813	−0.250	−0.478	0.090	0.730 **	0.712 **	0.531 **	−0.235 **	0.673 **	—

Note: ** *p* < 0.01; ACC = Accessibility, PC = Privacy Concern, EMP = Perceived Empathy, PU = Perceived Usefulness, PA = Perceived Anthropomorphism, TRU = Trust, BI = Behavioral Intention.

**Table 6:** R^2^, Adjusted R^2^, and Inner VIF Values of the Structural Model.

Endogenous Construct	Predictor	Inner VIF	R^2^	Adjusted R^2^
BI	ACC	1.030	0.660	0.657
	PC	1.097		
	PU	1.919		
	TRU	2.002		
EMP	PA	1.000	0.350	0.348
PU	ACC	1.009	0.464	0.461
	TRU	1.009		
TRU	EMP	1.002	0.551	0.549
	PC	1.002		

Note: ACC = Accessibility, PC = Privacy Concern, EMP = Perceived Empathy, PU = Perceived Usefulness, PA = Perceived Anthropomorphism, TRU = Trust, BI = Behavioral Intention.

**Table 7:** Direct effects (*n* = 375).

Hypothesis		Standard β	f^2^	SD	|t|	*p*-Value	Support
H1:	ACC → BI	0.334	0.320	0.033	10.174	<0.001	YES
H2a:	ACC → PU	0.102	0.019	0.040	2.556	0.011	YES
H2b:	PU → BI	0.115	0.020	0.046	2.493	0.013	YES
H3a:	PA→ EMP	0.591	0.538	0.034	17.590	<0.001	YES
H3b:	EMP → TRU	0.703	1.098	0.024	28.681	<0.001	YES
H4:	PC → BI	−0.086	0.020	0.032	2.724	0.006	YES
H5a:	PC → TRU	−0.209	0.097	0.033	6.306	<0.001	YES
H5b:	TRU → BI	0.601	0.531	0.043	14.051	<0.001	YES
H6:	TRU → PU	0.664	0.815	0.029	22.842	<0.001	YES

Note: ACC = Accessibility, PC = Privacy Concern, EMP = Perceived Empathy, PU = Perceived Usefulness, PA = Perceived Anthropomorphism, TRU = Trust, BI = Behavioral Intention.

**Table 8:** Specific indirect effects.

Path	β	Bootstrap SE	Lower	Upper	Result
ACC → PU → BI	0.012	0.007	0.0011	0.0274	Weakly Supported ^a^
PA → EMP → TRU	0.416	0.032	0.3500	0.4738	Supported
PC → TRU → BI	−0.126	0.023	−0.1748	−0.0858	Supported
PA → EMP → TRU → BI	0.250	0.027	0.1963	0.3045	Supported
PA → EMP → TRU → PU → BI	0.032	0.013	0.0060	0.0582	Supported
PC → TRU → PU → BI	−0.016	0.007	−0.0302	−0.0031	Supported
TRU → PU → BI	0.076	0.031	0.0145	0.1380	Supported

Note: ^a^ Statistical inference for indirect effects was based on the 95% percentile bootstrap confidence intervals. The *p*-values are based on the corresponding t-statistic approximation and are reported for completeness. An indirect effect was considered statistically significant when its 95% percentile bootstrap confidence interval did not include zero. ACC = Accessibility, PC = Privacy Concern, EMP = Perceived Empathy, PU = Perceived Usefulness, PA = Perceived Anthropomorphism, TRU = Trust, BI = Behavioral Intention.

## Data Availability

The data presented in this study are available on request from the corresponding author.

## Appendix A

**Items**		**Source**
**Accessibility**		
ACC1	I can use AI-based mental health services without restrictions of time and place.	(Wu & Wang, 2025)
ACC2	The process of accessing and using AI-based mental health services is convenient and easy to operate.	
ACC3	Overall, I can conveniently access and use AI-based mental health services.	
**Privacy Concern**		
PC1	I am concerned that the personal information I provide to AI may be used for purposes to which I have not consented.	(Niu et al., 2026)
PC2	I am concerned that AI may collect too much personal information.	
PC3	I often think twice before providing personal information to AI.	
PC4	I am concerned that AI lacks sufficient measures to protect my personal information.	
**Perceived Empathy**		
EMP1	I believe that generative artificial intelligence can understand my feelings and thoughts.	(Niu et al., 2026)
EMP2	I believe that generative artificial intelligence can truly care about and value me.	
EMP3	I believe that generative artificial intelligence can respond appropriately to my emotions.	
**Perceived Usefulness**		
PU1	Using AI-based mental health tools can help me relieve negative emotions more quickly.	(Sharma et al., 2025)
PU2	Using AI-based mental health tools can improve the effectiveness of my emotional regulation.	
PU3	Using AI-based mental health tools can make it easier for me to regulate my mental state.	
PU4	Using AI-based mental health tools can improve my psychological adjustment performance.	
PU5	Using AI-based mental health tools can help me save time and effort in emotion management.	
**Perceived Anthropomorphism**		
PA1	The responses from AI are natural, and I do not feel that they are artificial.	(Balakrishnan et al., 2022; Li et al., 2025; Sharma et al., 2025)
PA2	Communicating with AI psychological counseling feels like communicating with a real person.	
PA3	When using AI psychological counseling, I can feel a warm emotional atmosphere.	
TRU1	I believe that generative artificial intelligence is trustworthy.	(Niu et al., 2026)
TRU2	The information provided by generative artificial intelligence is credible.	
TRU3	I believe that generative artificial intelligence platforms are trustworthy.	
TRU4	I believe that the responses generated by these platforms are accurate and reliable.	
TRU5	I am willing to trust the psychological counseling advice provided by generative artificial intelligence.	
**Behavioral Intention**		
BI1	I intend to use AI psychological counseling tools in the future to cope with my psychological distress.	(Wijaya et al., 2025)
BI2	I plan to use AI psychological counseling tools frequently.	
BI3	If given the choice, I would be willing to use AI psychological counseling to help myself.	
BI4	I would prioritize AI psychological counseling as a form of psychological support.	
BI5	I would recommend AI psychological counseling tools to others.	
